# *E. coli*-Produced BMP-2 as a Chemopreventive Strategy for Colon Cancer: A Proof-of-Concept Study

**DOI:** 10.1155/2012/895462

**Published:** 2012-01-19

**Authors:** Saravanan Yuvaraj, Sa'ad H. Al-Lahham, Rajesh Somasundaram, Patrick A. Figaroa, Maikel P. Peppelenbosch, Nicolaas A. Bos

**Affiliations:** Department of Cell Biology, University Medical Center Groningen, University of Groningen, A. Deusinglaan 1, 9713 AV Groningen, The Netherlands

## Abstract

Colon cancer is a serious health problem, and novel preventive and therapeutical avenues are urgently called for. Delivery of proteins with anticancer activity through genetically modified bacteria provides an interesting, potentially specific, economic and effective approach here. Interestingly, bone morphogenetic protein 2 (BMP-2) is an important and powerful tumour suppressor in the colon and is thus an attractive candidate protein for delivery through genetically modified bacteria. It has not been shown, however, that BMP production in the bacterial context is effective on colon cancer cells. Here we demonstrate that transforming *E. coli* with a cDNA encoding an ileal-derived mature human BMP-2 induces effective apoptosis in an *in vitro* model system for colorectal cancer, whereas the maternal organism was not effective in this respect. Furthermore, these effects were sensitive to cotreatment with the BMP inhibitor Noggin. We propose that prevention and treatment of colorectal cancer using transgenic bacteria is feasible.

## 1. Introduction

**Figure 1:**
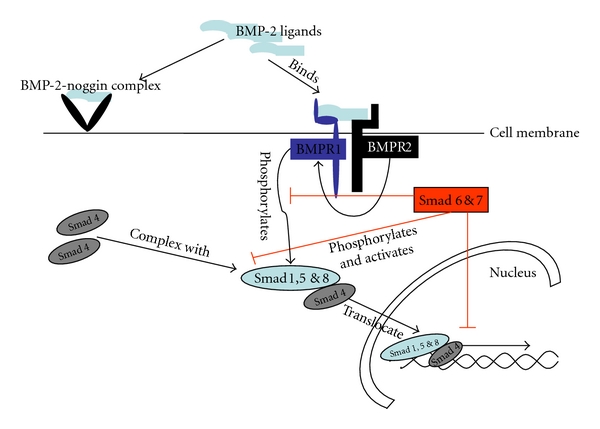
BMP binds to Type I (BMPR1A, BMPR1B) and Type II (BMPRII) serine threonine kinase transmembrane receptors and triggers a signal transduction cascade initiated via Smad family proteins. Signaling cascade through Smad take place via three Smad family proteins: receptor-mediated Smads.

Bone morphogenetic protein (BMP) is a member of the large TGF-*β* superfamily of morphogenetic ligands. Originally BMP was identified as a morphogen involved in bone formation, and later this protein emerged as an important signalling mediator during embryonic development and as a critical component of the morphogenetic code in derivatives of all three germ layers [1]. BMP signalling is antagonized by endogenous extracellular proteins, such as noggin, which bind BMPs and thus limit the extent of BMP signalling [2]. BMP binds to Type I (BMPR1A, BMPR1B) and Type II (BMPRII) serine-threonine kinase transmembrane receptors and triggers a signal transduction cascades mediated through the Smad cascade of signal transducers. Signalling through Smads takes place via three different classes of Smad family proteins: receptor-regulated Smads (Smad1, 5, and 8), co-Smad/Smad 4 (common mediator of Smad), and inhibitory Smads (Smad 6 and 7, negative regulators of Smad). Finally the BMP-Smad pathway activates direct or indirectly BMP target genes in the nucleus via cotranscriptional partners (Figure 1) [3]. Thus, BMP forms a complex with extracellular matrix proteins; for this reason its biological activity is confined to a local niche, tribute to its powerful morphogenetic action. 

BMP-2 has a crucial role during the embryonic development of digestive organs, for instance in stomach gland formation [4]. Expression is maintained through adulthood where it helps maintaining tissue homeostasis in this continuously regenerating organ [5], and its presence seems required to suppress transformation *in vivo, *for instance, following *Helicobacter* infection [6] or at the cancer-prone transition zones [7]. Likewise, also the colon is characterized by high expression of BMP-2 [6, 8, 9], where genetic loss of signalling components is intimately associated with the development of both sporadic cancer [10, 11] and with genetic polyposis syndromes, in particular juvenile polyposis [12] and also in gastric and colorectal cancer methylation of the BMP-2 promoter is a frequent event [13]. Especially important is also that the preventive action of statins on the development of colorectal cancer is mediated through the BMP pathway [14], producing epigenetic reprogramming and reducing colorectal cancer cell stemness [15, 16]. In apparent agreement, also in other parts of gastrointestinal tract, BMP signaling is intimately linked to the cancer process. BMP-2 is a negative regulator of hepatocyte proliferation downregulated in the regenerating liver [17], although in the oesophagus BMP-2 production may actually be implicated in induction of the precancerous condition Barrett's esophagus [18]. Together, these data indicate that BMP-2 acts as a powerful tumor suppressor in the columnar intestine and thus that application of exogenous BMP-2 may be useful in combating cancer cells. In agreement with such an application of BMP-2, Wen et al. have demonstrated that BMP-2 inhibited cell growth and induced cell differentiation in normal and cancerous gastric cell lines [19].

Colon cancer is an epithelial cancer which develops as a result of uncontrolled cellular proliferation and dysregulation of cellular apoptotic mechanisms [20], and its pathogenesis is undoubtedly related to the complex interaction of mucosal immunology with the microbiological ecology [21]. Conventional treatment for colon cancer such as surgery, radiotherapy, and chemotherapy has only limited efficacy and leads to serious side effects. Likewise current chemopreventive strategies only partially reduce risk, whereas endoscopic screening is expensive and unpopular with patients. Targeted therapies that by local application of anticancer molecules induce apoptosis in early phases of cancer development would evidently represent an important step forward. In this sense oral ingestion of genetically engineered bacteria may represent a golden bullet, as they by definition limit their action to the mucosa and are cheap to produce and apply to patients [22, 23]. The power of this strategy has been demonstrated in a variety of preclinical models [24–27] directed at combating mucosal inflammation using the production of immunomodulatory molecules, and the applicability of such a strategy for human disease was convincingly shown in a clinical trial of *Lactococcus lactis* producing interleukin-10 to treat Crohn's disease [28]. The applicability for preventing or treating early cancer has not been investigated, however. Evidently, however, mucosal delivery of the colonic tumour suppressor BMP-2 could prove highly interesting in this respect, but would require proof that BMP produced in the bacterial context is capable of counteracting colon cancer cells. BMP-2 has been produced from mammalian cell cultures and in plants as active form [29]. Demonstration of efficacy on colon cancer cells of bacterially produced BMP-2 would constitute an important step forward to come to applying transgenic bacteria for cancer prevention. Here we show that BMP-2 can be successfully expressed by *E. coli* and that such bacteria can kill cancer cells in a noggin-sensitive fashion, providing proof of principle for this strategy.

## 2. Methods and Materials

### 2.1. DLD-1 Culture

DLD cells were cultured at 37°C under a humidified 95% O_2_/5% CO_2_ atmosphere. The attaching DLD cells were cultured in RPMI supplemented with 10% fetal calf serum according to routine procedures.

### 2.2. Cloning of Human BMP-2

**Figure 2:**
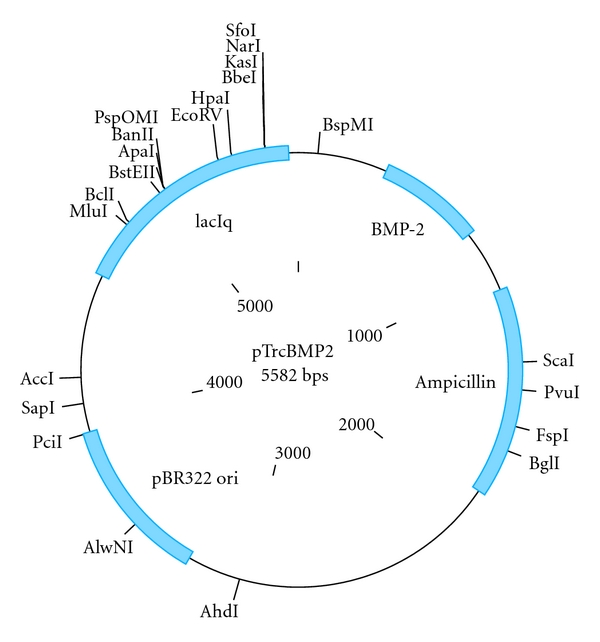
Schematic layout of the pTrcHis TOPO TA expression vector (Invitrogen, Netherland) used in this study for prokaryotic expression and the strategy employed to insert the ileal-derived human BMP-2 in this vector. The same vector, but driving LacZ expression, was used as control organism.

The human BMP-2 cDNA sequence was amplified by PCR using the primer 5′ATTGCCGGCGACCCGCTGTCTTCTA′3 and 5′ATCGATGCGACACCCACAAC′3 from normal human ileum. The primers contained the *ClaI* and *NgoMIV* restriction sites for future cloning into *L. lactis*. The entire PCR product was cloned into the Topo pTris prokaryotic expression vector and transformed into *E. coli* K-12 strain (Figure 2). The new plasmid was named as pTrisBMP-2. Confirmation of cloning and transformation was done by PCR on *E. coli* colonies using above-mentioned primer to confirm the presence of human BMP-2 in the colonies. Next, restriction enzyme analysis was performed, using *Bam*HI and *Age*I, to confirm correct orientation of the insert (human BMP-2) into the pTrcHis TOPO TA expression vector. The expected size of a correct orientation of the insert in the backbone was 291 bp, and with a reversed orientation 1030 bp. Subsequently the positive colonies were sequenced and checked for correct reading frame.

### 2.3. Expression of BMP-2 in *E. coli*

The *E. coli* strain K-12 transformed with pTrisBMP-2 was cultured in LB medium containing ampicillin (50 *μ*g/mL) at 37°C; when the OD_600_ of culture reached 0.6, the expression of BMP-2 was induced by addition of IPTG to a final concentration of 1 mM and cultivated for 2 more hours [30].

### 2.4. Western Blotting

Expression of BMP-2 was confirmed by the western blotting [31]. The molecular weight of the protein was checked by SDS-PAGE, which present 44 kDa. For immunodetection, samples were separated by SDS-PAGE and electrotransferred to a nitrocellulose membrane. The membrane was blocked with 5% nonfat milk in PBS-T buffer (PBS containing 0.05% Tween 20) and then incubated with mouse anti-human BMP-2 polyclonal antibody (1 : 2000) and Rabbit anti-His tag (1 : 5000) antibodies for 2 hours. The membrane was washed three times with PBS-T buffer and then incubated in anti-mouse and anti-rabbit HRP-conjugated secondary antibody at 1 : 2000 dilution in PBS-T buffer for 1 hour. The membrane was washed three times with PBS-T buffer, and the expressed proteins were visualized using ECL plus western blotting detection system (Amersham).

### 2.5. Coculture of DLD Cells and *E. coli* and Caspase Activity Assay

Experiments to address the effects of BMP-2 produced from the prokaryotic expression vector on apoptosis of cancer cells were performed as follows: an overnight culture of BMP-2 containing *E. coli* was inoculated in LB-broth until the OD_600_ reached 0.6 and induced with 1 mM IPTG. The induced bacteria were incubated with a confluent DLD-1 cell culture (with 10% FCS) in 6-well plates for 5 hours. The cells were lysed, and the protein concentrations were estimated using BIO-Rad DC Protein Assay kit (Bio-Rad). Caspase-3 activity was assayed in cells using a fluorimetric kit (Promega) according to the manufacturer's instructions. Briefly, the proluminescent substrate containing the DEVD (sequences are in a single-letter amino acid code) is cleaved by caspase-3. After caspase cleavage, a substrate for luciferase (aminoluciferin) is released; this results in the luciferase reaction and the production of luminescent signal. An equal volume of reagents and 20 *μ*g/mL proteins from the treated DLD-1 cells were added to a white-walled 96-well plate and incubated at room temperature for 1 h. The luminescence of each sample was measured in a plate-reading luminometer [32–35].

## 3. Results

### 3.1. Construction of pTrisBMP-2

Although prevention of colorectal cancer through the delivery of tumour suppressive cytokines to cancer cells by transgenetic bacteria is an attractive approach, it has not yet been shown that such cytokines can influence cancer cells when produced in bacterial context. To address this conceptual issue, the human BMP-2 gene was amplified from cDNA derived from normal ileum by PCR. The 1088 bp product was successfully cloned into pTris as to obtain the pTrisBMP-2 vector, and using PCR and restriction analysis the plasmid which contained the BMP-2 gene in the correct orientation was selected. Subsequent sequence analysis revealed that the obtained BMP-2 was in the right reading frame with three mutations were observed where one led to a change in the amino acid. This mutation may be due to a Taq error or to an acquired mutation in the volunteer from which the BMP-2 gene was derived. The vector obtained lends it well for prokaryotic transformation, and subsequently experiments were initiated to which extent it also allows production of BMP through bacteria.

### 3.2. BMP-2 Expression in *E. coli*

**Figure 3:**
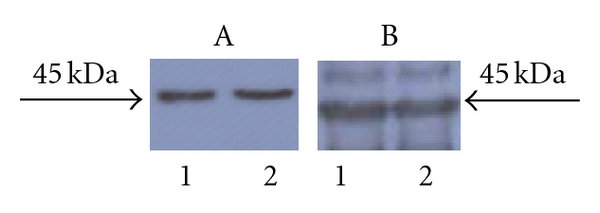
Illustration of expression of the transgene. Western blot analysis of BMP-2 expression in transformed *E. coli.* Panel A shows analysis of employing a poly-His tag antibody after 1 hr of IPTG induction (labelled 1) and after 3 hrs of IPTG induction (labelled 2). Panel B shows analysis of employing an anti-BMP-2 antibody after 1 hr of IPTG induction (labelled 1) and after 3 hrs of IPTG induction (labelled 2).

Small-size cultures (10 mL) of the positive clones of pTrisBMP-2 were subjected to IPTG induction to identify clones capable of expressing high levels of the recombinant protein. Ten clones showed expression of the unique predicted 44 kDa protein after IPTG induction. The BMP-2 production increased in parallel with the duration of induction. Without IPTG induction, two clones did not show the expression of this protein as confirmed by SDS-PAGE. The expression of the BMP-2 was further confirmed by western blot using anti-BMP-2 and anti-His tag antibody (Figure 3). We concluded that we can efficiently express BMP-2 through a bacterial vector and that the resulting bacterial clones would allow testing of whether production of the tumour suppressive cytokine in this bacterial context has anticancer activity. 

### 3.3. Apoptosis Assay

**Figure 4:**
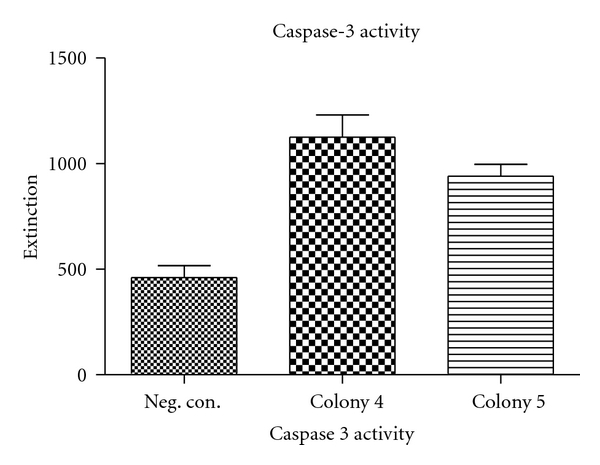
Illustration of expression anticancer activity of transformed bacteria on colon cancer cultures. BMP-2 expressing colonies were co-cultured with DLD-1 cells. Caspase-3 activity was measured using a fluorescent detection methodology. Colonies 4 and 5 show a significant higher caspase-3 activity compared to the negative control. Statistical significance was confirmed using a heteroscedastic two-sided Student's *t*-test. In this figure the negative control was bacteria containing a LacZ gene.

*E. coli* colonies were cocultured with DLD-1 cells for different time periods, and afterwards the potential and colon cancer activity of the bacterial on the cancer cells were assessed using caspase-3 activity as pseudo-endpoint. Importantly, control bacteria which express the LacZ gene (which we assume to be inert) do not provoke marked apoptosis in colorectal cancer cultures; thus bacteria *per se* do not seem to exert significant activity in this respect. Colon cancer cultures, however, co-cultured with bacteria containing the BMP-2 gene exhibited significant caspase-3 activity, testimony of a potent anticancer activity (Figure 4). Although caspase-3 is a relatively late marker in the apoptotic process, evident activation of the cysteine protease was clearly visible already after 5 hrs of coculture. The specificity of the effect is demonstrated by its sensitivity to the specific BMP antagonist noggin (data not shown). We conclude that tumour-suppressive cytokines can be produced through prokaryotes and retain efficacy in the bacterial context. Thus, we propose that mucosal delivery of tumour-suppressive cytokines is feasible.

## 4. Discussion

The clinical trail utilising IL-10 expressing *L. lactis* for the treatment of Crohn's disease has confirmed that such bacteria represent a viable approach for the mucosal release of therapeutically exciting proteins. This has opened new avenues to explore and use transgenic bacteria as a vehicle for delivery of this type of target molecules locally and as a consequence avoid the unwanted side effects which occur when such therapeutics are administered systemically. Whether this approach was also feasible for the delivery of tumour-suppressive cytokines remained unclear. Especially the capacity of prokaryotic organisms to produce functionally relevant amounts of such molecules and whether these molecules would retain efficacy when delivered to cancer cells in the bacterial context remained unexplored. Here we show large amounts of BMP-2 expressed by *E. coli*. Furthermore, we report a potential approach for inducing colon cancer cells death by apoptosis mediated by the recombinant BMP-2 protein produced by this *E. coli*. In recent years, a better perceptive of pathogenic mechanisms has provided novel targets and strategies for colon cancer therapy. These vary from novel chemotherapeutic agents, therapeutic antibodies, to target small molecules involved in the signal transduction. The successful clinical trails to treat patients with IBD using *L. lactis* producing IL-10 now opens the window to deliver anticancer agent via oral therapy with recombinant bacteria. Thus transgenic bacteria may become a vehicle to deliver associated target molecules locally and consequently avoid unwanted side effects associated with systemic administration.

BMP-2 expressed in *E. coli* shows clear proapoptotic effects when cocultured with DLD-1 cells. Although *E. coli* does not have the same posttranslation modification system as that present in humans, the normal vector for intestinal BMP-2 production, the BMP-2 produced by this bacterium, is evidently biologically active, and thus differences in prokaryotic and eukaryotic protein processing can be overcome. Recently many signalling pathway mediators involved in cancer have been identified which are promising targets for cancer therapy. These molecules should be delivered specifically to the cancer cells by targeting to tumour cells. Based on these requirements, we developed a recombinant human BMP-2 molecule. Addition of noggin which is antagonistic to BMP-2 inhibited apoptosis, further illustrating specificity. *In total*, our experiments provide proof of concept that mucosal delivery of therapeutic proteins is feasible, and the promising results obtained further encourage to continue by expressing the recombinant BMP-2 on the surface of *L. lactis* which would represent a next step in quest to combat colon cancer through transgenic bacteria.
